# Antiproliferative effects of masitinib and imatinib against canine oral fibrosarcoma *in vitro*

**DOI:** 10.1186/s12917-016-0712-x

**Published:** 2016-06-04

**Authors:** Milan Milovancev, Stuart C. Helfand, Kevin Marley, Cheri P. Goodall, Christiane V. Löhr, Shay Bracha

**Affiliations:** Department of Clinical Sciences, College of Veterinary Medicine, Oregon State University, Corvallis, OR 97331 USA; Department of Biomedical Sciences, College of Veterinary Medicine, Oregon State University, Corvallis, OR 97331 USA

**Keywords:** Dog, Oral fibrosarcoma, Masitinib, Imatinib, Platelet-derived growth factor receptor

## Abstract

**Background:**

Canine oral fibrosarcoma (COF) is one of the most common oral tumors in dogs and carries a guarded prognosis due to a lack of effective systemic therapeutic options. Mastinib and imatinib are two commonly used tyrosine kinase inhibitors (TKIs) in veterinary oncology but their potential efficacy against COF is uncharacterized. To begin investigating the rationale for use of these TKIs against COF, the present study tested for the presence TKI targets PDGFR-α, PDGFR-β, Kit, and VEGFR-2 and examined the *in vitro* effects on cell viability after TKI treatment alone or with doxorubicin.

Immunohistochemistry for PDGFR-α, PDGFR-β, Kit, and VEGFR-2 was performed in 6 COF tumor biopsies. Presence of these same receptors within 2 COF cell lines was probed by reverse transcription-polymerase chain reaction and, for those with mRNA detected, confirmed via western blot. Effects on cell viability were assessed using an MTS assay after masitinib or imatinib treatment alone (0-100 μM), or in combination with doxorubicin (0-3000 nM doxorubicin). Anti-*PDGFRB* siRNA knockdown was performed and the effect on cell viability quantified.

**Results:**

Expression of the TKI targets evaluated was similar between the 2 COF cell lines and the 6 COF tumor biopsies: PDGFR-α and PDGFR-β were detected in neoplastic cells from most COF tumor biopsies (5/6 and 6/6, respectively) and were present in both COF cell lines; *KIT* and *KDR* were not detected in any sample. Masitinib and imatinib IC50 values ranged from 7.9–33.4 μM, depending on the specific TKI and cell line tested. The addition of doxorubicin resulted in synergistic cytotoxicity with both TKIs. Anti-*PDGFRB* siRNA transfection reduced PDGFR-β protein expression by 77 % and 67 % and reduced cell viability by 24 % (*p* < 0.0001) and 28 % (0 = 0.0003) in the two cell lines, respectively.

**Conclusions:**

These results provide rationale for further investigation into the use of TKIs, possibly in combination with doxorubicin, as treatment options for COF.

## Background

Canine oral fibrosarcoma (COF) is one of the three most common oral neoplasms in dogs [1]. Compared to other anatomic locations, COF exhibits a biologically aggressive behavior with recurrence rates following resection of 24–59 %, metastasis in up to 30 % of cases, and reported median survival times of 7–24 months [2–9]. The most recent of these studies included 65 dogs and found significant predictors of median survival time to include tumor location (maxillary location better than mandibular), size (smaller tumors better), type of surgery (aggressive surgery better than conservative), histologic margin status, and grade (low grade better). This study included 14 dogs that received adjuvant systemic therapy (4 received doxorubicin and 10 received metronomic chemotherapy) but because of this low sample size and the fact that therapy was often initiated after relapse of disease, no conclusions could be drawn regarding the potential efficacy of this treatment strategy [8]. Currently, the prognosis for this disease remains guarded due to a lack of effective systemic therapeutic options to address potential metastasis as well as local recurrence [1–9].

The use of receptor tyrosine kinase inhibitors (TKIs) for targeted therapy in veterinary oncology is increasing as indicated by the growing number of clinical reports [10–15]. Although some reports describe use of TKIs alone, others have reported on observed clinical efficacy when combined with traditional cytotoxic chemotherapeutic agents and/or piroxicam [10, 11, 13, 15, 16]. Proposed mechanisms behind combination therapy include chemosensitization as well as immunomodulatory effects such as suppression of regulatory T cells and restoration of T cell-mediated immune responses [16]. Masitinib is conditionally approved by the United States Food and Drug Administration and the European Medicines Agency for use against canine mast cell tumors. Masitinib targets PDGFR-α and -β, Kit, Lyn, and to a lesser degree, the FGFR3 and FAK pathways [16]. Masitinib may also affect VEGFR-2 levels [14]. Imatinib is another TKI that targets some of the same kinases as masitinib, including PDGFR-α, PDGFR-β, and Kit [16, 17]. Although not approved by the United States Food and Drug Administration for use in veterinary patients, off-label veterinary use of imatinib has been reported with favorable results in canine and feline cancer patients [18–21].

To our knowledge, there are no reports that have profiled expression of tyrosine kinases in COF, nor the potential for targeting by masitinib or imatinib. The purpose of this study was to (1) evaluate the expression of PDGFR-α, PDGFR-β, Kit, and VEGFR-2 in archived COF biopsies and immortalized cell lines and (2) assess the effects on cell viability of two TKIs (masitinib and imatinib), either alone or in combination with doxorubicin, against the cell lines *in vitro*. The results presented herein begin to shed light on this strategy as a potential future therapy for COF.

## Results

### Archived canine oral fibrosarcoma tumors express PDGFR-α and –β protein

**Fig. 1:**
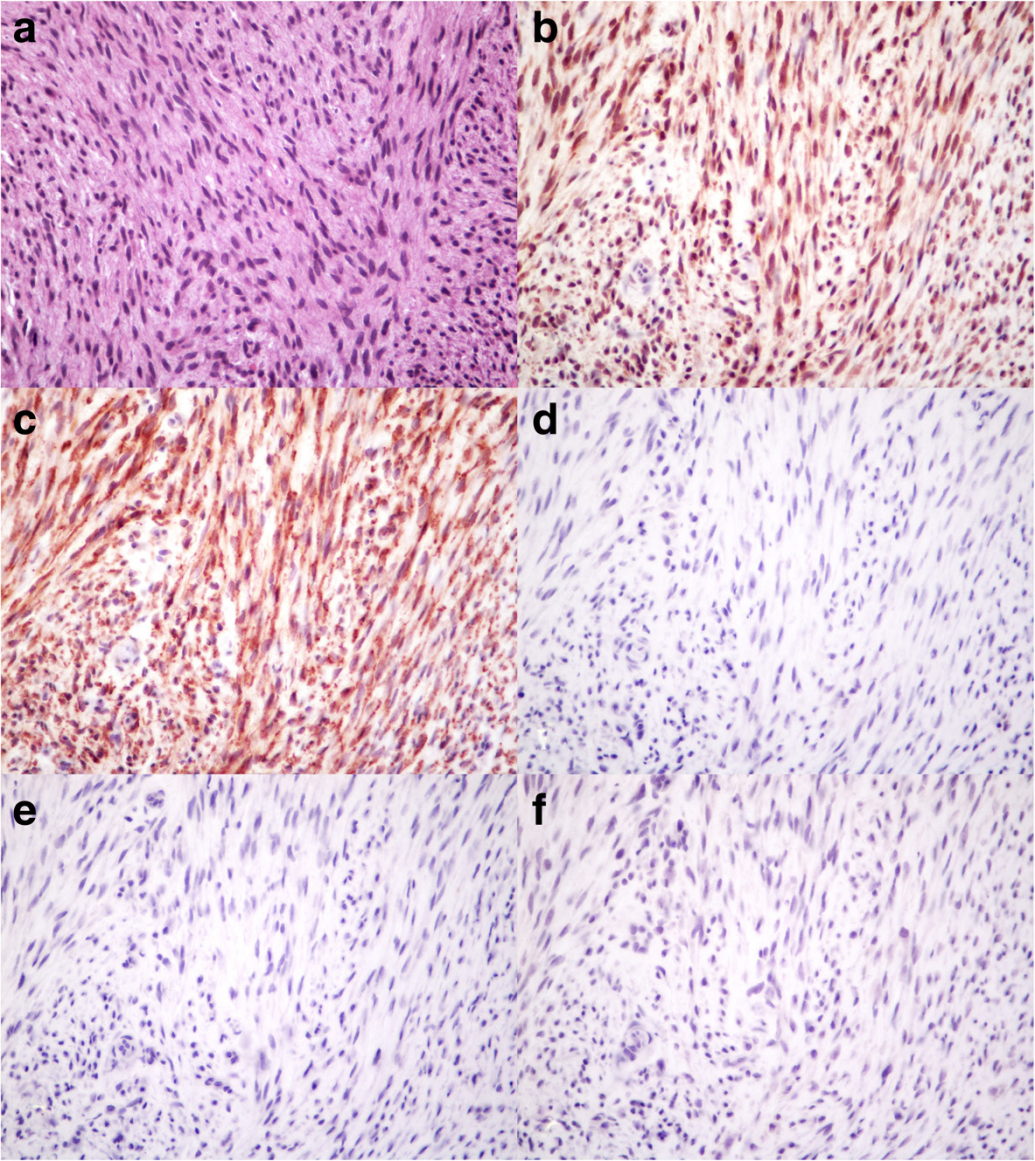
Immunohistochemistry of archived canine oral fibrosarcoma tumor biopsies. Representative photomicrographs of an archived canine oral fibrosarcoma tumor specimen (dog #4) stained with (**a**) hematoxylin and eosin, **b** PDGFR-α immunohistochemistry, **c** PDGFR-β immunohistochemistry, **d** Kit immunohistochemistry, **e** VEGFR-2 immunohistochemistry, and **f** rabbit negative control; 400×. Immunoreactivity for PDGFR-α (both cytoplasmic and nuclear locations) and PDGFR-β (predominantly cytoplasmic location) is visible. No staining is seen for VEGFR-2, Kit, or in the rabbit negative control

**Fig. 2:**
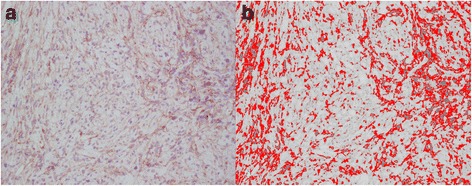
Semi-quantitative assessment of immunoreactivity via image threshold-processing. Representative photomicrograph of PDGFR-β immunohistochemical staining of an archived canine oral fibrosarcoma tumor specimen (dog #6) before (**a**) and after (**b**) threshold-processing for semi-quantitative assessment of immunoreactivity; 400×. Red pixels are reported as percentage of total image pixels to provide a semi-quantified measure of immunoreactivity

Immunohistochemistry (IHC) for PDGFR-α, PDGFR-β, Kit, and VEGFR-2 demonstrated differential expression of each protein amongst the six archived tumor specimens with good agreement between subjective observer-derived assessments and semi-quantitative software-derived results (Table 1). Representative photomicrographs for each of the proteins evaluated are shown in Fig. 1. A representative software threshold-processed image of tumor cell immunoreactivity for PDGFR-β is shown in Fig. 2. Higher percentages represent more immunoreactivity (i.e. pixels above the user-defined threshold for IHC stain). Mitotic counts in five of six sarcomas were low, ranging from 1 to 4 in ten 400x high power fields. Case 3 had a much higher mitotic count (*n* = 15). This tumor also had the largest nuclei, poorest overall organization, and most intense IHC staining for both PDGFRs.

**Table 1:** Immunohistochemistry reactivity scores

Protein	Dog #	% Cells	Location	Intensity	% Area
PDGFR-α	1	75	C, N	+	29.4
	2	80	C, N	+	26.2
	3	100	C, N	++	21.6
	4	90	C, N	++	43.9
	5	0	–	–	0
	6	90	C, N	+	11.6
PDGFR-β	1	90	C, M	+++	34.8
	2	70	C, M	++	40.8
	3	100	C	+++	38.1
	4	60	C	+++	23.8
	5	90	C	+	19.6
	6	80	C	++	21.8
Kit	1	0	–	–	0
	2	0	–	–	0
	3	0	–	–	0
	4	0	–	–	0
	5	0	–	–	0
	6	0	–	–	0
VEGFR-2	1	0	–	–	0
	2	0	–	–	0
	3	0	–	–	0
	4	0	–	–	0
	5	0	–	–	0
	6	0	–	–	0

Subjective scoring of immunoreactivity of 6 archived canine oral fibrosarcoma cases for VEGFR-2, PDGFR-α, PDGFR-β, and Kit. The estimated percentage of tumor cells displaying immunoreactivity, the predominant location(s) of staining (C = cytoplasmic, M = membranous, or N = nuclear), and the subjective intensity of staining (+, ++, or +++) are displayed along with semi-quantitative measurement of immunoreactivity using computer image analysis software and threshold-processed photomicrographs

Staining for PDGFR-α was detected in the cytoplasm and nuclei of 75–100 % of neoplastic cells in five of the six tumor samples, with a relatively uniform staining intensity among samples. In all sections, PDGFR-α staining was also present in the cytoplasm and nuclei of endothelial cells including neoplastic endothelial cells of a canine metastatic hemangiosarcoma control sample. Similarly, PDGFR-β was detected in the cytoplasm of 60–100 % of neoplastic cells in all six tumor samples. There was relatively uniform intensity and subcellular location of immunostaining of neoplastic cells in five of the biopsy samples with a similar distribution but weaker staining intensity in the remaining sample. Two samples also showed cell membrane associated PDGFR-β staining. PDGFR-β stained the cytoplasm of endothelial cells in all sections including neoplastic endothelial cells in the hemangiosarcoma control sample. VEGFR-2 and Kit staining were uniformly negative in neoplastic cells of all six COF tumor samples. Endothelial cells around but not within the tumors had cytoplasmic staining for VEGFR-2, whereas neoplastic cells of a metastatic hemangiosarcoma did not stain. Most sections had interstitial mast cells that displayed a largely membrane-associated staining pattern for Kit. In the mast cell tumor control sample, most neoplastic cells had cytoplasmic, perinuclear, punctate staining for Kit (pattern 2); less than 5 % showed diffuse cytoplasmic staining (pattern 3) [22].

### Canine oral fibrosarcoma cell lines express PDGFR-α and –β at both mRNA and protein levels

**Fig. 3:**
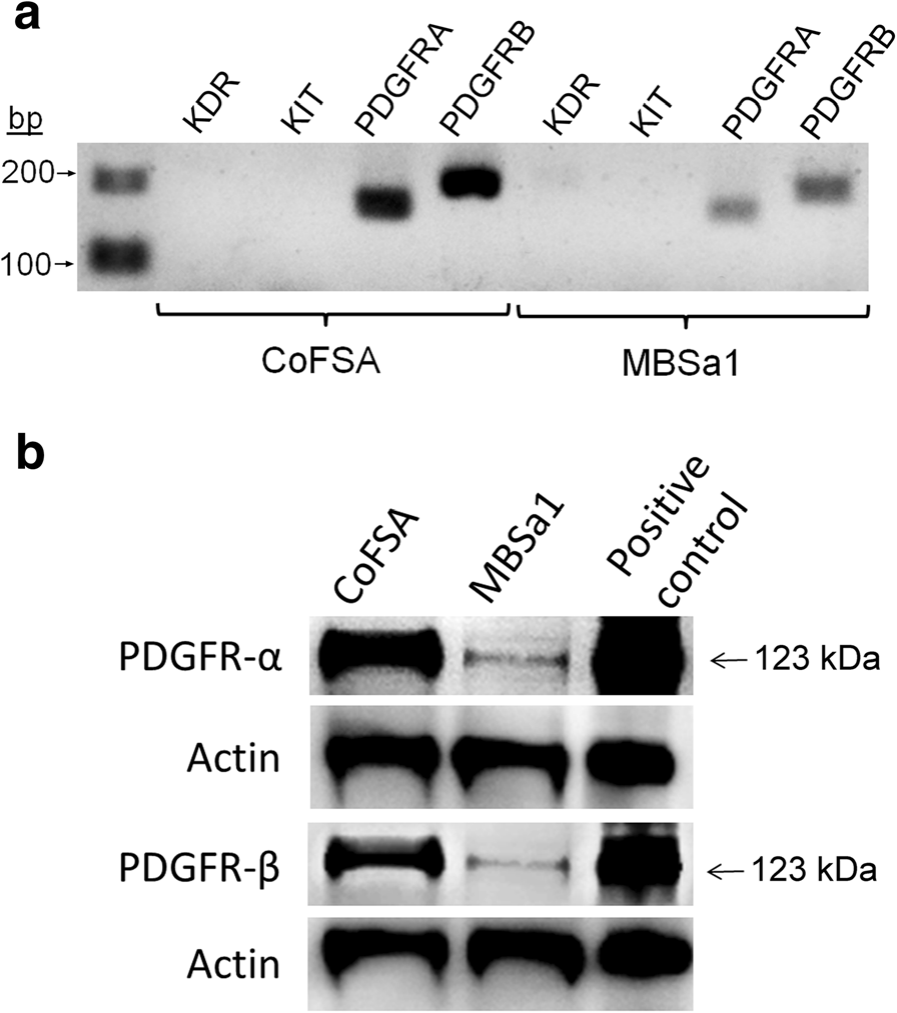
Receptor tyrosine kinase expression in cell lines. **a** Reverse transcriptase-polymerase chain reaction for *KDR*, *KIT*, *PDGFRA*, and *PDGFRB* in CoFSA and MBSa1 cell lines demonstrates presence of transcript for both *PDGFRA and PDGFRB* at the expected amplicon size with no evidence of *KDR* or *KIT* transcript. The molecular weight ladder is shown on the left side of the image with the base pairs (bp) listed. **b** Western blot of PDGFR-α and PDGFR-β demonstrating protein presence in both CoFSA and MBSa1 cell lines at the expected molecular weight of 123 kDa. A lysate from 293 T cells was used as a positive control (SC-114235, Santa Cruz Biotechnology, Dallas, TX)

*PDGFRA* and *PDGFRB* mRNA was reverse transcribed and amplified from exponentially growing MBSa1 and CoFSA cells by reverse transcription-polymerase chain reaction (RT-PCR; Fig. 3a). Amplicons were of the predicted size and sequencing reaction results matching the published sequence with 100 % homology. Transcripts for *KIT* and *KDR* were not detected (Fig. 3a) despite using two different canine-specific primer sets.

Western blots showed strong expression of both PDGFR-α and –β in cell lysates from CoFSA, with weaker expression in MBSa1 (Fig. 3b). These data coincide with the apparent mRNA signals shown in these two cell lines (Fig. 3a).

### Masitinib or imatinib alone, or in combination with doxorubicin, inhibit canine oral fibrosarcoma cell viability

**Fig. 4:**
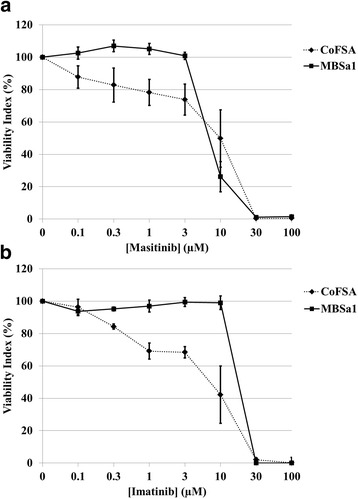
Cell viability after treatment with masitinib or imatinib. Graphical plot of the effects of **a** masitinib and **b** imatinib on viability of MBSa1 and CoFSA cells. Cell viability was assessed using a MTS assay of MBSa1 and CoFSA treated with escalating concentrations of masitinib or imatinib after 72 h of incubation. Masitinib treated cells displayed decreased viability relative to the vehicle-treated control at 10.0, 30.0, and 100.0 μM for both MBSa1 and CoFSA (*p* < 0.0001). Imatinib treated cells displayed decreased viability relative to the vehicle-treated control at 30.0 and 100.0 μM for MBSa1 (*p* < 0.0001) and at 1.0, 3.0, 10.0, 30.0, and 100.0 μM for CoFSA (*p* < 0.0001). Plotted values are mean ± standard error of the mean. The calculated IC50 of masitinib for MBSa1 and CoFSA is 9.1 and 12.0 μM, respectively. The calculated IC50 of imatinib for MBSa1 and CoFSA is 33.4 and 7.9 μM, respectively

Masitinib treated cells displayed decreased viability relative to the vehicle-treated control at concentrations of 10, 30, and 100 μM for both MBSa1 and CoFSA cell lines (*p* < 0.0001; Fig. 4a). The calculated IC50 of masitinib for MBSa1 and CoFSA is 9.1 and 12.0 μM, respectively. Imatinib treated cells displayed decreased viability relative to the vehicle-treated control at 30.0 and 100.0 μM for MBSa1 (*p* < 0.0001) and at 1.0, 3.0, 10.0, 30.0, and 100.0 μM for CoFSA (*p* < 0.0001; Fig. 4b). The calculated IC50 of imatinib for MBSa1 and CoFSA is 33.4 and 7.9 μM, respectively.

**Fig. 5:**
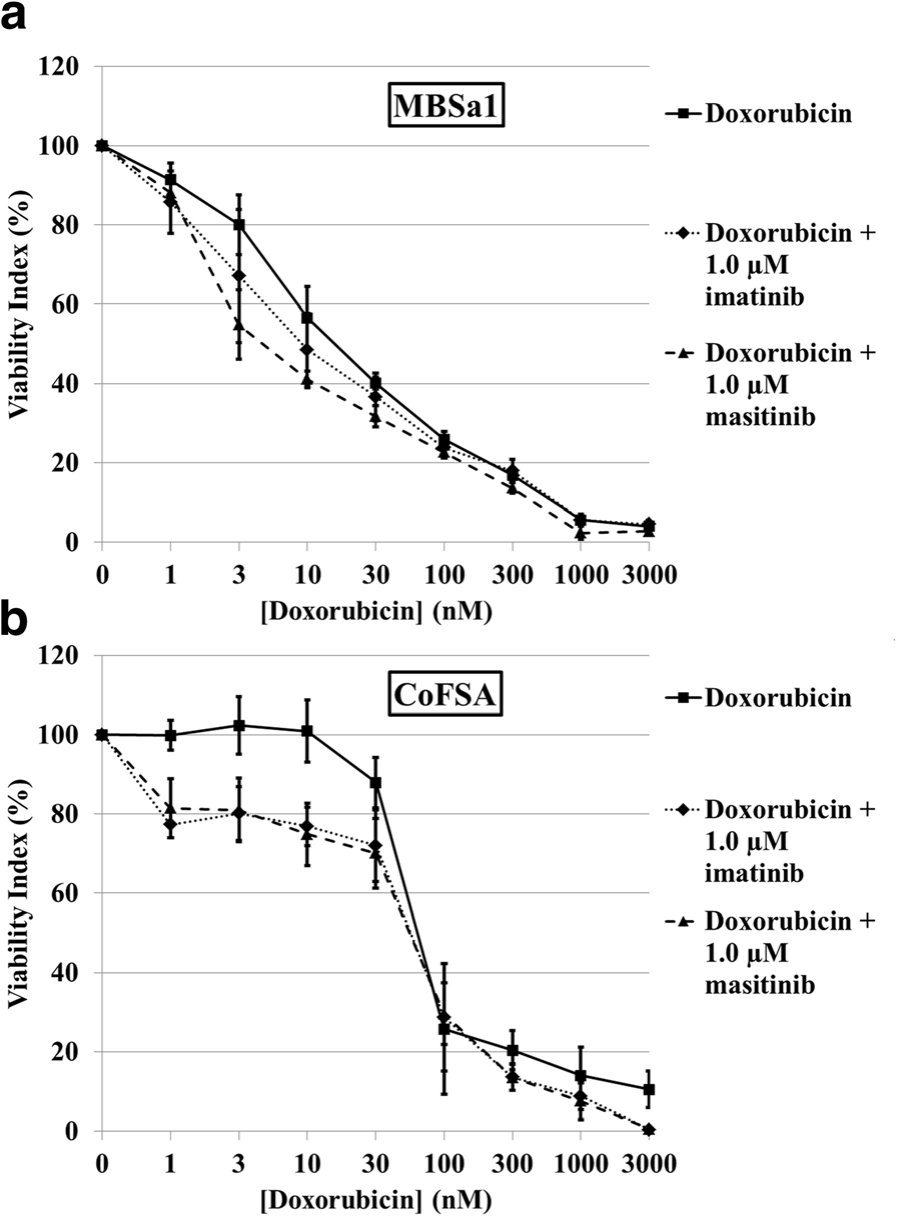
Cell viability following doxorubicin treatment alone or combined with 1.0 μM of either masitinib or imatinib. Graphical plot of the effects of treatment with escalating concentrations of doxorubicin alone or combined with 1.0 μM of either masitinib or imatinib on viability of (**a**) MBSa1 and (**b**) CoFSA cells. Cell viability was assessed using a MTS assay following treatment with the above drug concentrations after 72 h of incubation. Masitinib showed a synergistic interaction with doxorubicin at all concentrations for MBSa1 and at 10, 300, 1000, and 3000 nM for CoFSA. Imatinib showed a synergistic interaction with doxorubicin at 1, 3, 10, 30, and 100 nM for MBSa1 and at 300, 1000, and 3000 nM for CoFSA. Plotted values are mean ± standard error of the mean. Synergism was defined as being present when the surviving fraction of cells exposed to the combination of doxorubicin and either tyrosine kinase inhibitor was lower than the product of the surviving fraction of cells exposed to the tyrosine kinase inhibitor alone multiplied by the surviving fraction of cells exposed to doxorubicin alone. See Materials and Methods section for detailed synergism calculation methods

The combination of either 1.0 μM masitinib or imatinib with doxorubicin yielded synergistic reductions in cell viability for both cell lines (Fig. 5). Combination treatment demonstrated synergism in MBSa1 cells at all doxorubicin concentrations for masitinib and at 1, 3, 10, 30, and 100 nM doxorubicin concentrations for imatinib (Fig. 5a). Due to the greater reduction in cell viability seen with 1.0 μM of either TKI alone in CoFSA cells, synergism was shown only at the highest doxorubicin concentrations tested: 10, 300, 1000, and 3000 nM for masitinib and 300, 1000, and 3000 nM for imatinib (Fig. 5b).

### PDGFRB siRNA knocks down PDGFR-β protein expression and reduces oral fibrosarcoma cell line viability

**Fig. 6:**
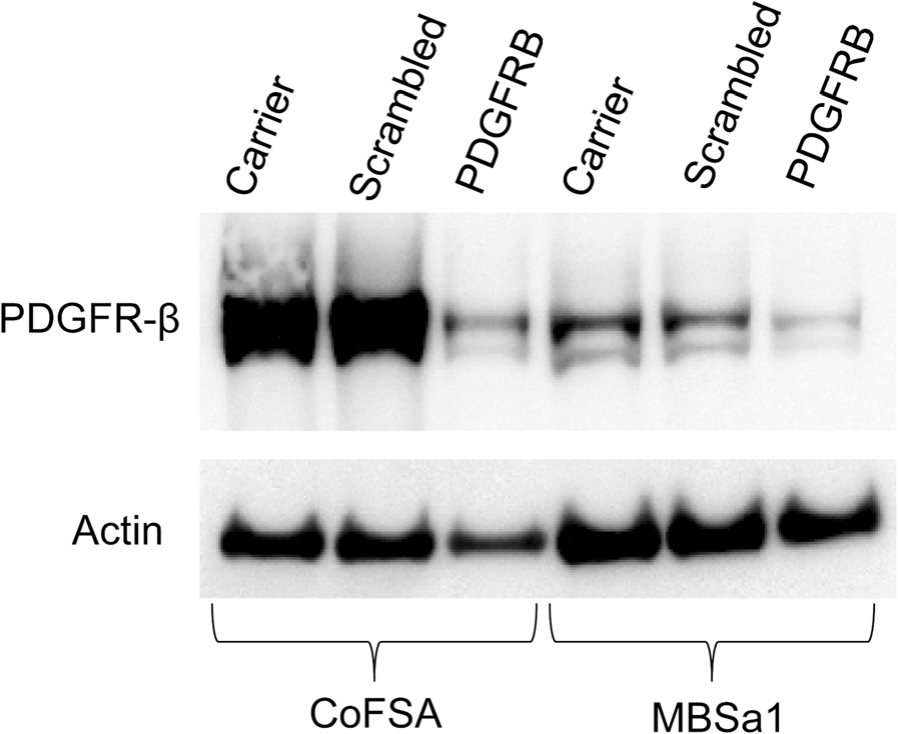
PDGFR-β reduction following *PDGFRB* siRNA transfection. Effect of *PDGFRB* siRNA transfection on PDGFR-β expression in CoFSA and MBSa1 cells assessed via western blot. Reduced PDGFR-β levels are represented as decreased band intensity in the *PDGFRB* siRNA treated lanes for both cell lines. Cells were incubated with siRNA for 48 h, as described in methods. Scrambled siRNA sequence used to account for nonspecific, off-target effects. To account for differences in protein loading between lanes, final PDGFR-β knockdown was reported as percentage of actin-normalized PDGFR-β band intensity in the siRNA treated lane relative to actin-normalized PDGFR-β band intensity in the control (vehicle-treated) lane using computer image analysis software with a gel analysis package (ImageJ v1.47, NIH, Bethesda, MD)

Western blot analysis demonstrated that PDGFR-β is expressed in both MBSa1 and CoFSA cell lines, with a marked reduction in PDGFR-β expression evident in both cell lines after *PDGFRB* siRNA transfection (Fig. 6). Densitometry measurements of actin-normalized PDGFR-β band intensity (expressed as a percentage of the vehicle-only treated control cells) revealed a reduction of PDGFR-β protein in MBSa1 and CoFSA cells of 77.4 % and 67.4 %, respectively.

**Fig. 7:**
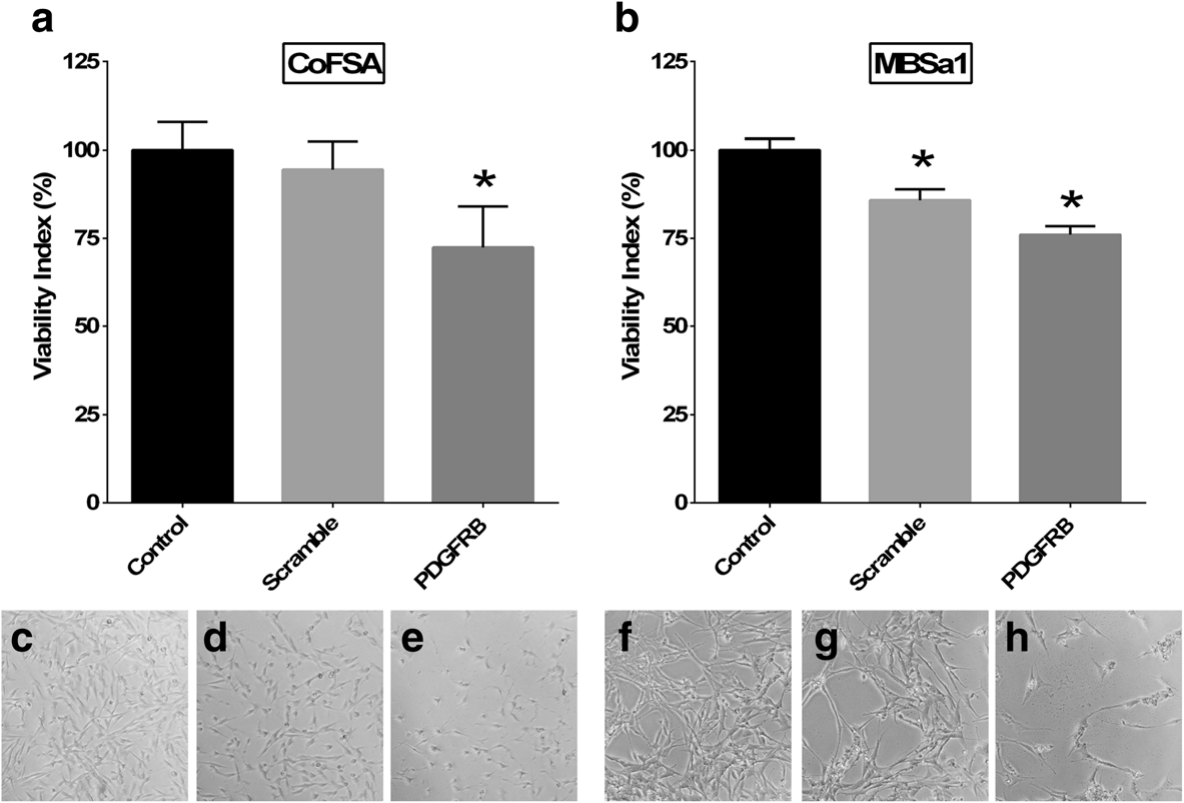
Cell viability following *PDGFRB* siRNA transfection. Effect of *PDGFRB* siRNA transfection on (**a**) CoFSA and (**b**) MBSa1 cell viability assessed via an MTS assay after 72 h of incubation, with representative photomicrographs of cells under each condition (CoFSA cells treated with (**c**) vehicle alone, **d** scrambled siRNA sequence, and **e** *PDGFRB* siRNA; MBSa1 cells **f** treated with vehicle alone, **g** scrambled siRNA sequence, and **h** *PDGFRB* siRNA). Scrambled siRNA sequence used to account for nonspecific, off-target effects. “*” indicates statistically significant (*p* < 0.05) differences in cell viability compared to vehicle-treated controls. Because siRNA transfection was performed during two independent experiments, statistical analysis for these data was performed using the 6 replicates within one representative siRNA experiment. Visual assessment of cellular appearance in *PDGFRB* siRNA treated samples (**e** and **h**) display apoptotic bodies and marked cellular morphologic deterioration

Cell viability after *PDGFRB* siRNA transfection was significantly reduced in both MBSa1 (mean reduction of 24.0 %; *p* < 0.0001) and CoFSA (mean reduction of 27.6 %; *p* = 0.0003) cell lines compared to vehicle-treated control cells (Fig. 7). Visual comparison of siRNA-transfected cells to control cells revealed a greater negative effect on cell viability than was reflected by the MTS assay results (Fig. 7).

### Effect of masitinib and imatinib, alone or combined with doxorubicin, on oral fibrosarcoma cell line caspase activity

**Fig. 8:**
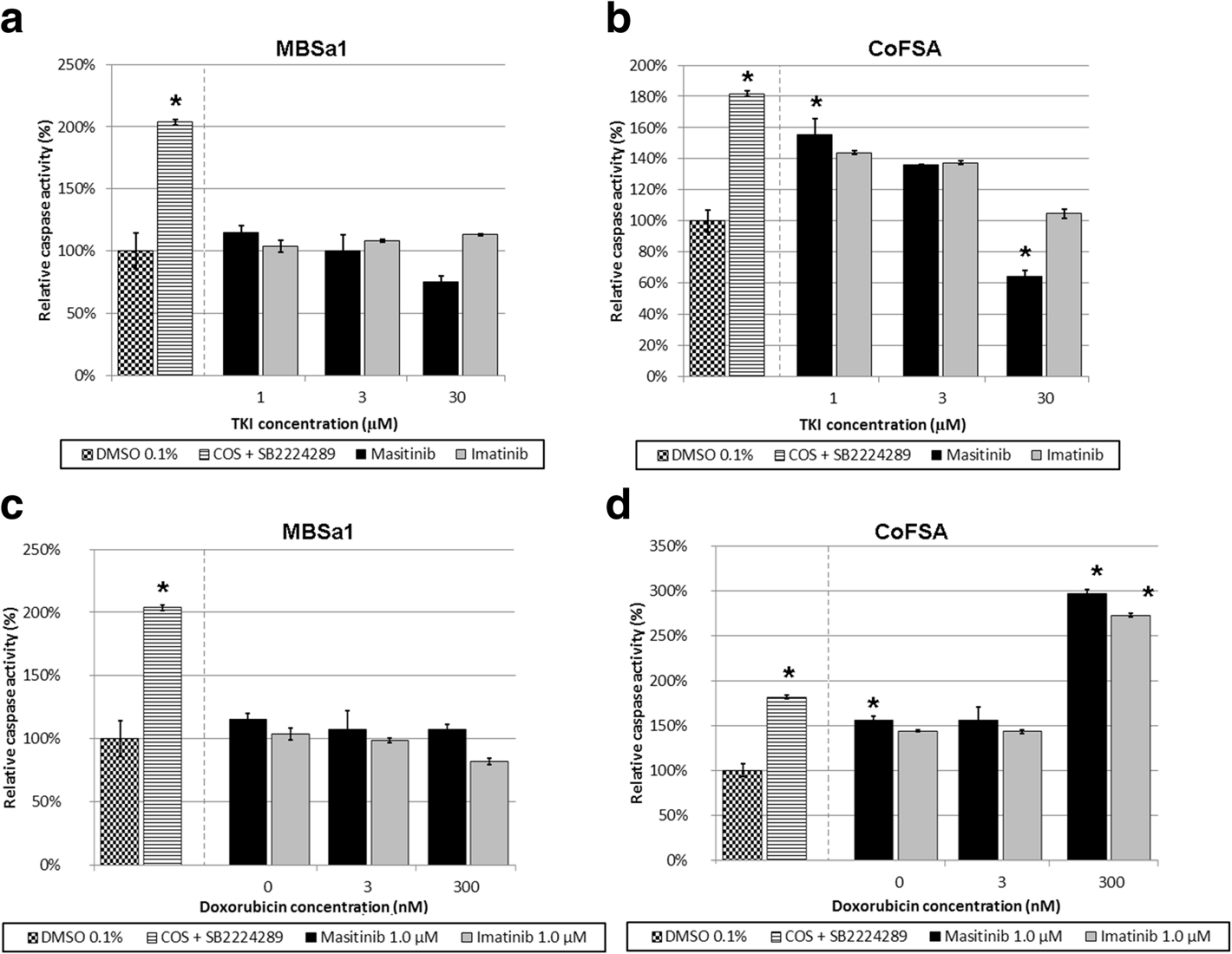
Cell apoptosis following treatment with imatinib and masitinib, alone or combined with doxorubicin. Graphical plot of relative caspase activity following treatment with escalating concentrations of masitinib and imatinib alone (**a** and **b**) and doxorubicin combined with 1.0 μM of either masitinib or imatinib (**c** and **d**) on MBSa1 and CoFSA cells, respectively. Caspase activity was assessed using a luminogenic caspase-3/7 substrate assay following treatment with the above drug concentrations after 72 h of incubation and expressed as a percentage of caspase activity within vehicle-treated (DMSO 0.1 %) control cells. COS cells treated with SB2224269 represent a positive control. Plotted values are mean ± standard error of the mean. “*” indicates statistically significant (*p* < 0.001) difference compared to the vehicle-treated controls indicated by a one-way ANOVA with Dunnett’s correction

MBSa1 cells did not demonstrate significant changes in caspase-3/7 activity at any drug concentration tested (Fig. 8a and c). In contrast, CoFSA cells did display significantly increased caspase activity at 1.0 μM masitinib alone (Fig. 8b) and at 300 nM doxorubicin combined with either 1.0 μM masitinib or imatinib (Fig. 8d); CoFSA cells showed significantly reduced caspase activity following treatment with 30 μM masitinib alone (Fig. 8b).

## Discussion

This study begins to explore the potential rationale for using two commonly prescribed TKIs (masitinib and imatinib) as adjunctive treatment in COF. The stimulus for this investigation is based on the premise that targeted small molecule therapy may provide an adjuvant therapeutic strategy for control of COF following surgery, given the challenge of obtaining complete surgical tumor excision and the reluctance of many pet owners to pursue adjuvant radiotherapy. We began by testing for the presence of targets of these two TKIs, including PDGFR-α, PDGFR-β, VEGFR-2, and Kit, in six archived COF tumors and two immortalized COF cell lines. Our results demonstrate a similar expression profile between the immortalized cell lines and the archived tumor samples, leading into the second aim of the study: assessing the effects of the two TKIs on cell viability, either alone or in combination with doxorubicin. Our data show both cell lines were relatively resistant to single-agent TKI treatment, with substantial reductions in cell viability and an increase in apoptotic activity being seen only at relatively high concentrations in most experiments. Both TKIs met the criteria for synergistic *in vitro* cytotoxicity when combined with doxorubicin, although the magnitude of this effect was relatively small. Cumulatively, the present study provides insight into the potential validity of future *in vivo* investigations exploring the use of TKIs, possibly in combination with traditional cytotoxic chemotherapeutic agents, as adjuvant treatment options in COF.

Of the four potential TKI targets evaluated via IHC, PDGFR-β was the most consistently expressed (6/6 of archived tumors) and showed the strongest immunoreactivity across tumor samples. Furthermore, PDGFR-β was detected within both cell lines at the mRNA and protein levels. PDGFR-α was also frequently detected with 5/6 archived tumor samples showing immunoreactivity, but with lower subjective staining intensity and scoring lower on our semi-quantitative immunoreactivity measurements. Both cell lines also expressed PDGFR-α at both mRNA and protein levels. The COF tumor sample with the highest mitotic count and least degree of differentiation was among the tumors with the most intense IHC staining for PDGFR-β and PDGFR- α, consistent with the positive effect of PDGF on cell proliferation. Neither Kit nor VEGFR-2 were detected at the protein level within tumor samples or at mRNA levels within the cell lines. These data indicate that at least two targets of the TKIs used in this study are present within COF tumors and provided a rationale for proceeding with the evaluation of cell viability following TKI treatment.

The *in vitro* effect of the tested TKIs on CoFSA and MBSa1 cell viability was observed to be relatively similar between the two drugs. These observations are consistent with the shared targets between the two TKIs [15, 16]. MBSa1 cells were consistently resistant to either TKI, with significant cytotoxic effects seen only at high concentrations and no significant changes in apoptosis elicited by any drug concentration tested. By comparison, CoFSA cells were similarly resistant to masitinib but slightly more sensitive to imatinib, with a modest but statistically significant reduction in cell viability at ≥ 1 μM concentrations. CoFSA cells also demonstrated significant increases in apoptosis at select drug concentrations, consistent with their increased sensitivity to the treatments evaluated in this study. CoFSA cells also showed a significant decrease in relative caspase activity at 30 μM masitinib, which may reflect a paucity of cells with caspase activity due to the extremely low cell viability at this TKI concentration, as supported by our MTS assay results. These relatively minor differences in TKI sensitivity between cell lines may reflect a combination of differences in drug targets between the two TKIs tested along with differences in the cell lines used [14, 16]. To the authors’ knowledge, no studies have characterized specific receptor tyrosine kinase pathway dependence in either MBSa1 or CoFSA cell lines. Several other *in vitro* veterinary studies have reported similarly high masitinib or imatinib IC50 concentrations in canine hemangiosarcoma and feline injection site sarcoma cell lines [12, 14, 23]. In contrast, the cell-based IC50s for masitinib against PDGFR-α, PDGFR-β, and Kit have been reported as 0.3, 0.05, and 0.15 μM, respectively, in an IL3-dependent hematopoietic cell line [24]. These values are markedly lower than the IC50 values seen in the present and prior *in vitro* veterinary studies, raising the possibility that the observed reductions in cell viability may be due to off-target effects. This is also compatible with the calculated IC50 values from another veterinary study evaluating *in vitro* masitinib effects on a variety of immortalized canine cancer cell lines [25]. The reason for this relative resistance to single-agent TKI treatment in the cell lines in the present report, as well as in the those cell lines used in the referenced studies, is not fully understood but likely reflects the cell lines’ lack of dependence on the targeted pathways for survival [12, 14, 23, 25]. However, as pointed out in previous reports, findings such as described in the current study do not preclude a potential clinical benefit of masitinib therapy either as a single agent targeting tumor-related angiogenesis, or perhaps more importantly, as a potential chemosensitizer [25].

To begin to investigate the potential role of either masitinib or imatinib as chemosensitizers in COF, we performed MTS assays using a range of doxorubicin concentrations, with or without 1.0 μM of either TKI. This concentration of TKI was chosen because pharmacokinetic studies in healthy Beagle dogs have shown that a clinically-relevant oral dose of 10 mg/kg of masitinib results in a serum maximum concentration of 1.3–1.5 μM [26]. Our data support an *in vitro* synergistic effect of either TKI with doxorubicin in both cell lines, although the effect was modest. The potential for myelosuppression, or other side-effects, may be increased when TKIs are combined with traditional cytotoxic chemotherapeutic agents *in vivo,* as shown in a study evaluating the safety of toceranib combined with vinblastine in dogs with mast cell tumors [16]. Maximal sensitization factors, potential alterations in drug pharmacokinetics, and alternative methods for determining pharmacologic synergism were not considered as they extend beyond the scope of the present study, but may form the basis for future investigations.

As PDGFR-β was found to be the most uniformly expressed receptor tyrosine kinase for TKI targeting in the present study, we chose to further investigate the contribution of PDGFR-β signaling to viability of COF cell lines. Knockdown of PDGFR-β protein expression via siRNA transfection was successful in both cell lines. The effect of this PDGFR-β protein reduction was associated with a significant reduction in cell viability in both cell lines as well as visibly apparent degenerative changes in cellular morphology. This suggests that PDGFR-β signaling plays a partial role in maintaining viability of COF cells, but the overall significance of this single signaling pathway to COF cell survival requires further investigation.

Although a thorough discussion of the mechanism of action of imatinib and masitinib is beyond the scope of this report, a few select points are worth highlighting. Both TKIs are considered small molecule inhibitors that selectively interfere with specific receptor tyrosine kinase activity (PDGFR-α, -β, and Kit; masitinib also targets Lyn, the FGFR3 and FAK pathways and possibly VEGFR-2 levels) [14, 16, 27]. Through occupying the receptor’s active site, thereby blocking receptor tyrosine kinase phosphorylation, TKIs prevent subsequent activation of downstream pathways. Depending on the cell’s dependence on the targeted pathways, this may result in cell death [16]. Some TKIs, such as masitinib, have shown an anticancer action that extends beyond inhibition of its primary targets, and may include disruption of additional signaling pathways associated with tumor progression, metastasis, and chemoresistance [25, 26, 28, 29]. The *in vivo* tumor microenvironment is characterized by varying levels of hypoxia and acidity, which influence tumor cell behavior and drug sensitivity, potentially rendering them more or less sensitive to TKI treatment [30]. PDGFR is emerging as a key regulator of mesenchymal cells within the tumor microenvironment of many common human malignancies [31]. Blockade of PDGFR signaling has been shown to reduce metastasis in *in vivo* murine models of colorectal and prostate cancers [32–34]. These points serve to illustrate some of the impetus behind this study’s investigations into the use of TKIs as a potential treatment strategy, although their applicability to imatinib and/or masitinib treatment of COF remain unknown at this time.

The primary limitations of the present study center on its *in vitro* nature and the limited experimental methods used. This study tested for receptor tyrosine kinase expression but did not evaluate receptor phosphorylation status (i.e. activation), receptor over- or under-expression, or effect of ligand stimulation on the receptors present. The presence of TKI targets does not necessarily imply their requirement for cell survival or a gain-of-function structural aberration conferring malignant behavior. This study evaluated effects of TKI treatment on COF cell line viability using an MTS assay, supplemented with an apoptosis assay for select drug concentrations, representing only a partial evaluation of potential TKI effects. Examples of additional treatment effects that could be examined in future studies include COF cell migration and/or metastasis. Finally, it is difficult to extrapolate *in vitro* results of TKI treatment to the far more complex *in vivo* scenario that includes interactions with the tumor microenvironment and the host immune system.

## Conclusions

In conclusion, this study identified expression of PDGFR-α and –β in COF tumor biopsies and cell lines. Treatment with masitinib or imatinib yielded *in vitro* reductions in cell viability which was enhanced synergistically by the addition of doxorubicin. Furthermore, the tested COF cell lines exhibited partial PDGFR-β dependency for survival. Taken together, these data support further investigation into the potential use of TKIs, potentially in combination with doxorubicin, to augment existing treatment options for COF.

## Methods

### Immunohistochemistry of archived canine oral fibrosarcomas

Medical records from dogs seen at the Oregon State University Lois Bates Acheson Veterinary Teaching Hospital between 2007 and 2011 were searched to identify histologically confirmed COF tumor biopsies. All tumors were comprised of elongate to spindle cells arranged in streams or bundles and whorls that produced variable amounts of collagenous matrix. Four of the tumors had small heterochromatic nuclei, case 1 had medium-sized and case 3 had large euchromatic nuclei. The diagnosis of COF was confirmed by examination of a representative hematoxylin and eosin stained section from each biopsy by a single board-certified veterinary anatomic pathologist (CVL). Serial sections 4–5 μm thick from paraffin-embedded formalin-fixed tumor biopsies were mounted on positively charged slides for IHC analysis of PDGFR-α, PDGFR-β, Kit, and VEGFR-2 expression using anti-human receptor-specific polyclonal rabbit antibodies (detailed in Table 2) [35–37].

**Table 2:** Antibodies and conditions used for immunohistochemical staining of archived canine oral fibrosarcoma tumor specimens

Target	Manufacturer	Antibody	Dilution	Species	HTAR
VEGFR-2	Novus Biologicals, Littleton, CO	NBP1-74001	1:100	Rabbit	+
PDGFR-α	Santa Cruz Biotechnology, Dallas, TX	SC-338	1:200	Rabbit	+
PDGFR-β	BioGenex Laboratories, San Ramon, CA	N463-UC	1:200	Rabbit	+
Kit	Dako North America, Carpinteria, CA	A4502	1:500	Rabbit	+

*HTAR* High temperature antigen retrieval at pH 6

High temperature antigen retrieval was performed with a microwave pressure cooker using Dako Target Retrieval solution (pH 6, 10 mins) according to the manufacturer’s recommendations^a^. IHC staining was performed on a Dako Autostainer (Dako North America, Carpinteria, CA) at room temperature (21 °C) after blocking for 10 mins with 3 % H_2_O_2_ (Sigma Laboratories, Santa Fe, NM) in TBST (Biocare Medical, Concord, CA) followed by Dako serum-free protein block (Dako North America, Carpinteria, CA) for 10 mins. The primary antibodies were diluted in Dako antibody diluent (Dako North America, Carpinteria, CA) and applied for 30 mins. Conditions and manufacturer information are detailed in Table 2. Specific antibody binding was detected using MaxPoly-One polymer HRP rabbit (ImmunoBioScienceIH-8064-custom-OrSU, ImmunoBioScience, Mukilteo, WA) for 10 mins followed by Nova Red (SK-4800, Vector Laboratories, Burlingame, CA) for 5 mins. Hematoxylin (Dako North America, Carpinteria, CA) diluted 1:3 in distilled water for 5 mins was used as a counter stain. Washes between steps were performed using TBST (Biocare Medical, Concord, CA), except no wash was performed for the protein block. Dako Universal Negative Control-Rabbit (Dako North America, Carpinteria, CA) was used as the negative control. Peritumoral non-neoplastic tissues were used as internal positive (endothelium or mast cells) and negative (epidermis) controls, a canine cutaneous mast cell tumor submitted as biopsy served as positive control for Kit staining, and a canine metastatic hemangiosarcoma (liver, kidney, testis, spleen) collected during necropsy was used as a positive control [37–39]. Evaluation of IHC staining for specificity was performed by a board-certified veterinary anatomic pathologist (CVL).

### Immunohistochemistry scoring

Immunoreactivity for PDGFR-α, PDGFR-β, Kit, and VEGFR-2 were scored by three of the investigators (CVL, MM, and SB) with the results representing a consensus agreement between the observers. The criteria evaluated included: percentage of tumor cells displaying immunoreactivity (assessed from a representative 40× field, after examining the slide in its entirety), the predominant location of staining (cytoplasmic, membranous, or nuclear), and relative visual intensity of staining (+, ++, or +++).

In addition, semi-quantitative measurement of immunoreactivity for the same proteins (using a photomicrograph of the same IHC field as described above) was carried out using a computer image analysis software package (ImageJ v1.47, NIH, Bethesda, MD) as previously described [40]. Output data recorded was the percentage of image pixels above the user-defined threshold to capture immunoreactivity.

### Cell lines and reagents

Two immortalized COF cell lines were tested: MBSa1 (provided by Dr. Marlene Hauck, North Carolina State University, Raleigh, NC, USA) and CoFSA (provided by Dr. Melanie Wergin, University of Zurich, Zurich, Switzerland). Both cell lines were derived from biopsies acquired from clinically affected dogs presented for spontaneously arising COF [41, 42]. Cells were cultured in RPMI-1640 medium supplemented with 10 % fetal bovine serum, 2 mM glutamine, 2 mM sodium pyruvate, 2 mM HEPES, and 1 % pen-strep in a humidified 5 % CO_2_ atmosphere at 37 °C.

Masitinib powder (provided by AB Science, Paris, France) was suspended in DMSO and stored at -80 °C until use. Imatinib was purchased from a commercial supplier (LC Labs, Woburn, MA), suspended in DMSO and stored at -80 °C until use. Doxorubicin HCl (2 mg/ml) in isotonic solution was purchased from a commercial supplier (Amneal-Agila, Glasgow, KY). Dimethyl sulofoxide concentrations in all experiments never exceeded 0.3 %.

### Reverse transcription-polymerase chain reaction

Expression of transcripts for *PDGFRA*, *PDGFRB*, *KIT*, and *KDR* was assessed in MBSa1 and CoFSA cells using RT-PCR. Cells were seeded into six-well plates (3 × 10^5^/well) suspended in 2.0 mL supplemented medium and allowed to adhere overnight. The cells were rinsed in PBS and RNA was isolated (RNeasy, Qiagen, Valencia, CA) and reverse transcribed to cDNA (High Capacity Reverse Transcription, Applied Biosystems, Foster City, CA) according to the manufacturers’ instructions. Targets were amplified from cDNA using the specific primers (Invitrogen, Carlsbad, CA) listed in Table 3 with the following accession numbers: [*PDGFRA* GenBank: XM532374.5; *PDGFRB* GenBank: NM001003382.1; *KIT* GenBank: XM005627969.2; and *KDR* GenBank: NM001048024.1]. Reverse transcription-polymerase chain reaction was performed according to standard methods [43] using Taq DNA polymerase (Invitrogen, Carlsbad, CA), with an annealing temperature of 58 °C, melting temperature of 94 °C, and run for 34 cycles on a thermocycler (Bio-rad Laboratories, Hercules, CA). Products were separated by agarose gel electrophoresis and visualized under ultra-violet light with propidium iodide and recorded using an Image Quant LAS4000 digital image capture system (GE Healthcare, Pittsburg, PA). Amplicons were purified using magnetic beads (Invitrogen, Carlsbad, CA) and sequenced on an ABI Prism 3730 Genetic Analyzer (Applied Biosystems, Grand Island, NY) using the Sanger method (BigDye Terminator v. 3.1 Cycle Sequencing Kit, Life Technologies, Grand Island, NY). Results reported are representative of three independent experiments, with each cell line tested in triplicate during each experiment.

**Table 3:** Primers used for reverse transcriptase-polymerase chain reaction of immortalized canine oral fibrosarcoma cell lines

Target	Forward primer sequence (5′-3′)	Reverse primer sequence (5′-3′)	Spanned mRNA sequence	Product size (bp)
*PDGFRA*	CCTCGATCCTTCCAAATGAA	GGTCACAAAAAGGCCACTGT	357-523	167
*PDGFRB*	GTGGTATGGGAACGGTTGTC	GTGGGATCTGGCACAAAGAT	228-421	194
*KIT*	CCCATTTAACCGAACGAGAA	TCTCCGTGATCTTCCTGCTT	2016-2226	211
*KDR*	GATCGGTGAGAAATCCCTGA	CTGGAAGTCATCCACGTTT	1266-1473	208

### Western blot

Western blots were performed against proteins for which mRNA transcripts were detected via RT-PCR in MBSa1 and CoFSA cells. Cells were seeded in 6-well plates (3 × 10^6^/well) and allowed to adhere overnight as described above. The cells were detached using a cell scraper, transferred to a micro-centrifuge tube, and centrifuged in a tabletop centrifuge (3 min, 1200 × g). Cell pellets were rinsed by re-suspending twice in 3 mL ice cold PBS and extracted in 50 μL ice cold RIPA buffer with protease and phosphatase inhibitor cocktail (Sigma Laboratories, Santa Fe, NM). Extracts were sonicated four times (1 s each) using a Model 150 T ultrasonic dismembrator (Fisher Scientific, Pittsburg, PA) and pelleted at 10,000 × g to remove cellular debris. Protein concentration was measured using a Bradford assay (Bio-rad Laboratories, Hercules, CA) according to the manufacturer’s instructions. Proteins (20 μg/lane) were separated on 4–12 % SDS polyacrylamide gels (Bio-rad Laboratories, Hercules, CA) and transferred to PVDF membranes. The membranes were blocked in 1.5 % bovine serum albumin and probed with either anti-PDGFR-β antibody (BioGenex, Fremont, CA) or anti-PDGFR-α antibody (antibody #SC-388, Santa Cruz Biotechnology, Dallas, TX) diluted 1:1000 and incubated overnight at 4 °C. The membranes were washed, probed with horseradish peroxidase-linked secondary antibody (SC-2005, Santa Cruz Biotechnology, Dallas, TX) diluted 1:20000, and exposed to substrate (ECL Select, GE Healthcare, Pittsburg, PA). A lysate from 293 T cells was used as a positive control (SC-114235, Santa Cruz Biotechnology, Dallas, TX). Bands were visualized and recorded using an Image Quant LAS4000 digital image capture system. Western blot results reported are representative of two independent experiments.

### Cell viability assay

An MTS colorimetric assay (CellTiter 96 Aqueous One Solution Cell Proliferation Assay, Promega, Madison, WI) was used according to the manufacturer’s instructions to assess the effects of masitinib and imatinib on viability of COF cell lines. Briefly, growing cells were seeded into 96-well plates at 2,500 cells/well suspended in 100 μL of supplemented medium and incubated overnight prior to adding drugs to allow adherence. Frozen aliquots of each TKI were thawed and diluted to twice the desired concentrations in supplemented medium prior to adding 100 μL to wells containing cells. The final TKI concentrations included the following: 0, 0.1, 0.3, 1.0, 3.0, 10.0, 30.0, and 100.0 μM. Cultures were maintained for 72 h following addition of the drugs, after which 150 μL of the media was removed and replaced with 20 μL MTS reagent premixed with 50 μL supplemented media. Cells were incubated in the presence of the MTS reagent for 2–4 h. Two cell-free, media-only wells were included in each experiment to generate assay background values, which were subtracted from the absorbance of each well prior to calculating viability indices. Results from TKI treated cells were compared against controls comprised of cells cultured under identical conditions with 0.3 % DMSO, but without added TKI. There was no difference in viability between cell lines treated with DMSO only (data not shown).

To assess the potential for a chemosensitizing effect of the tested TKIs with doxorubicin, MTS cell viability experiments were performed as described above with doxorubicin either alone or combined with 1.0 μM of either masitinib or imatinib. This concentration of TKI was chosen because pharmacokinetic studies in healthy Beagle dogs show that a clinically-relevant oral dose of 10 mg/kg of masitinib results in a serum maximum concentration of 1.3–1.5 μM [26]. Doxoribucin concentrations tested included 0, 1, 3, 10, 30, 100, 300, 1000, and 3000 nM. Results from drug-treated cells were compared to controls of cells cultured under identical conditions with 0.3 % DMSO and without added doxorubicin or TKI.

Three MTS experiments were performed independently and each condition was run in triplicate within each experiment.

The type of interaction between each TKI and doxorubicin was determined using the following equations [44]:

$$ \begin{array}{l}\mathrm{S}\mathrm{ynergistic} = {\mathrm{SF}}_{t+y} < {\mathrm{SF}}_t\mathrm{x}\ {\mathrm{SF}}_y\\ {}\mathrm{Additive} = {\mathrm{SF}}_{t+y} = {\mathrm{SF}}_t\mathrm{x}\ {\mathrm{SF}}_y\\ {}\mathrm{S}\mathrm{u}\mathrm{b}\hbox{-} \mathrm{additive} = {\mathrm{SF}}_t\mathrm{x}\ {\mathrm{SF}}_y < {\mathrm{SF}}_{t+y} < {\mathrm{SF}}_t\mathrm{and}\ {\mathrm{SF}}_y\\ {}\mathrm{Antagonistic} = {\mathrm{SF}}_{t+y} > {\mathrm{SF}}_t\mathrm{or}\ {\mathrm{SF}}_y\end{array} $$

SF_*t+y*_ = surviving fraction of cells exposed to the combination of either TKI and doxorubicin, SF_*t*_ = surviving fraction of cells exposed to either TKI alone, SF_*y*_ = surviving fraction of cells exposed to doxorubicin alone. These equations are appropriate provided the drug effect is reduced cell viability [45], a condition that was met at all drug concentrations except at 3 and 10 nM doxorubicin in CoFSA where the mean cell viability values for doxorubicin treatment alone were slightly increased. In these exceptions, the SF of the control group (i.e. cells, but no doxorubicin or TKI) was substituted in the equation for the SF of doxorubicin, as it was the more stringent condition for evaluating drug interaction.

All MTS testing was performed during three independent experiments, with each condition run in triplicate during each experiment.

### Inhibition of PDGFR-β expression by siRNA

PDGFR-β knockdown was used to assess the role of PDGFR-β in sustaining viability of COF cell lines MBSa1 and CoFSA. Cells were seeded into 96-well plates (5 × 10^3^ cells/well) and allowed to adhere overnight before transfection with a combination of four commercially purchased target-specific siRNAs (Life Technologies, Carlsbad, CA; 3 pmol per reaction) against canine *PDGFRB* (Dharmacon, Lafayette, CO) or a scrambled sequence (Mission siRNA Universal Negative Control #1, Sigma Laboratories, Santa Fe, NM) using Lipofectamine RNAiMAX (Invitrogen, Carlsbad, CA) according to the manufacturer’s directions. Cell viability was measured in 96-well plate format 72 h after siRNA transfection using the MTS colorimetric assay (CellTiter 96 Aqueous One Solution Cell Proliferation Assay, Promega, Madison, WI) as described above.

The *PDGFRB* siRNAs used were (5′–3′):

- Sequence #1: CCUUCAAGGUGGUGGUGAUTT
- Sequence #2: CCAUGAACGAACAGUUCUATT
- Sequence #3: GAAAUGAGGUGGUUAACUUTT
- Sequence #4: GAAUGACCAUCGAGAUGAATT

All siRNA data were normalized to the readouts taken from control cells treated with the transfection reagent alone, and included scrambled sequence controls to assess for nonspecific, off-target effects. Results from siRNA knockdown reported are representative of two independent experiments, with each condition run in sextuplicate during each experiment.

Western blot was repeated after siRNA treatment, as described above, using only the anti-PDGFR-β antibody(BioGenex, Fremont, CA). Quantification of siRNA PDGFR-β protein knockdown was performed using computer image analysis software with a gel analysis package (ImageJ v1.47, NIH, Bethesda, MD). PDGFR-β signal intensities in each lane were expressed as percentage of the total protein control (actin) loaded into respective lanes. Final PDGFR-β protein knockdown was reported as percentage of actin-normalized PDGFR-β band intensity in the siRNA treated lane relative to actin-normalized PDGFR-β band intensity in the control (vehicle-treated) lane.

### Apoptosis

A luminogenic caspase-3/7 substrate assay (Caspase-Glo 3/7, Promega, Madison, WI) was used to determine relative caspase activity in COF cell lines after TKI treatment, either alone or with doxorubicin. The drug concentrations tested were selected based on MTS results in order to represent the range of observed effects on cell viability. Cells were seeded in 96-well culture plates at a density of 2,500 cells/well and challenged with the following drugs (concentrations): masitinib (0, 1, 3, and 30 μM), imatinib (0, 1, 3, and 30 μM), masitinib 1.0 μM + doxorubicin (3 and 300 nM), and imatinib 1.0 μM + doxorubicin (3 and 300 nM). For an apoptosis positive control, COS cells were incubated with 6.25 μM SB2224289 (Tocris, Bristol, UK) [46]. For a vehicle control, cells were incubated with 0.1 % DMSO in media. Drugs were dissolved in DMSO, with cells exposed to a final vehicle concentration of 0.1 %. Cells were challenged for 72 h, after which the caspase-3/7 activity was quantified. The caspase activity assay was performed according to the manufacturer’s protocol. Cumulative luminescence over 1 s was measured using a luminometer (GloMax 96 Microplate Luminometer, Promega, Madison, WI). Relative caspase activity was calculated using the formula: relative caspase activity = (mean luminescence of treated cells)/(mean luminescence of vehicle control) × 100.

As this experiment was intended to act as a supplement to the MTS assay observations, only a single independent experiment (with each drug treatment condition in triplicate) was performed.

### Statistical analysis

Mean cell viability (as described above) was compared to vehicle-treated control cells using a one-way ANOVA with Dunnett’s multiple comparisons post-test. Except for siRNA experiments, all statistical analyses were performed using means from each independent experiment with standard deviations representing differences between the means. Because siRNA transfection was performed during two independent experiments, statistical analysis for these data were performed using the 6 replicates within one representative siRNA experiment. The 50 % inhibitor concentration (IC50) of masitinib and imatinib for CoFSA and MBSa1 were calculated using non-linear regression of the log of the inhibitor versus a variable slope response equation, with constraints set at 100 % for the top and 0 % for baseline. Relative caspase activities within the various drug treatment conditions were compared to vehicle-treated control cells with a one-way ANOVA with Dunnett’s correction using the technical replicate data from the apoptosis experiment. Significance was set at *p* < 0.05 and all statistical testing was performed using a commercially available computer software program (Graphpad Prism v6.02 for Windows, Graphpad Software, San Diego, CA).

## Abbreviations

COF, canine oral fibrosarcoma; TKIs, tyrosine kinase inhibitors; IHC, immunohistochemistry; RT-PCR, reverse transcription-polymerase chain reaction.

## References

[1] Liptak JM, Withrow SJ, Withrow SJ, Vail DM, Page RL (2013). Cancer of the gastrointestinal tract. Withrow & MacEwen’s Small Animal Clinical Oncology.

[2] Todoroff RJ, Brodey RS (1979). Oral and pharyngeal neoplasia in the dog: a retrospective survey of 361 cases. J Am Vet Med Assoc.

[3] Kosovsky JK, Matthiesen DT, Marretta SM, Patnaik AK (1991). Results of partial mandibulectomy for the treatment of oral tumors in 142 dogs. Vet Surg.

[4] Wallace J, Matthiesen DT, Patnaik AK (1992). Hemimaxillectomy for the treatment of oral tumors in 69 dogs. Vet Surg.

[5] Schwarz PD, Withrow SJ, Curtis CR, Powers BE, Straw RC (1991). Mandibular resection as a treatment for oral cancer in 81 dogs. J Am Anim Hosp Assoc.

[6] Schwarz PD, Withrow SJ, Curtis CR, Powers BE, Straw RC (1991). Partial maxillary resection as a treatment for oral cancer in 61 dogs. J Am Anim Hosp Assoc.

[7] Ciekot PA, Powers BE, Withrow SJ, Straw RC, Ogilvie GK, LaRue SM (1994). Histologically low-grade, yet biologically high-grade, fibrosarcomas of the mandible and maxilla in dogs: 25 cases (1982-1991). J Am Vet Med Assoc.

[8] Gardner H, Fidel J, Haldorson G, Dernell W, Wheeler B (2015). Canine oral fibrosarcomas: a retrospective analysis of 65 cases (1998-2010). Vet Comp Oncol.

[9] Frazier SA, Johns SM, Ortega J, Zwingenberger AL, Kent MS, Hammond GM (2011). Outcome in dogs with surgically resected oral fibrosarcoma (1997-2008). Vet Comp Oncol.

[10] Chon E, McCartan L, Kubicek LN, Vail DM (2012). Safety evaluation of combination toceranib phosphate (Palladia®) and piroxicam in tumour-bearing dogs (excluding mast cell tumours): a phase I dose-finding study. Vet Comp Oncol.

[11] de Vos J, Ramos Vega S, Noorman E, de Vos P (2012). Primary frontal sinus squamous cell carcinoma in three dogs treated with piroxicam combined with carboplatin or toceranib. Vet Comp Oncol.

[12] Lawrence J, Saba C, Gogal R, Lamberth O, Vandenplas ML, Hurley DJ (2012). Masitinib demonstrates anti-proliferative and pro-apoptotic activity in primary and metastatic feline injection-site sarcoma cells. Vet Comp Oncol.

[13] London C, Mathie T, Stingle N, Clifford C, Haney S, Klein MK (2012). Preliminary evidence for biologic activity of toceranib phosphate (Palladia®) in solid tumours. Vet Comp Oncol.

[14] Lyles SE, Milner RJ, Kow K, Salute ME (2012). In vitro effects of the tyrosine kinase inhibitor, masitinib mesylate, on canine hemangiosarcoma cell lines. Vet Comp Oncol.

[15] Robat C, London C, Bunting L, McCartan L, Stingle N, Selting K (2012). Safety evaluation of combination vinblastine and toceranib phosphate (Palladia®) in dogs: a phase I dose-finding study. Vet Comp Oncol.

[16] Bavcar S, Argyle DJ (2012). Receptor tyrosine kinase inhibitors: molecularly targeted drugs for veterinary cancer therapy. Vet Comp Oncol.

[17] Pardanani A, Tefferi A (2004). Imatinib targets other than bcr/abl and their clinical relevance in myeloid disorders. Blood.

[18] Kobayashi M, Kuroki S, Ito K, Yasuda A, Sawada H, Ono K (2013). Imatinib-associated tumour response in a dog with a non-resectable gastrointestinal stromal tumour harbouring a c-kit exon 11 deletion mutation. Vet J.

[19] Yamada O, Kobayashi M, Sugisaki O, Ishii N, Ito K, Kuroki S (2011). Imatinib elicited a favorable response in a dog with a mast cell tumor carrying a c-kit c.1523A>T mutation via suppression of constitutive KIT activation. Vet Immunol Immunopathol.

[20] Isotani M, Tamura K, Yagihara H, Hikosaka M, Ono K, Washizu T (2006). Identification of a c-kit exon 8 internal tandem duplication in a feline mast cell tumor case and its favorable response to the tyrosine kinase inhibitor imatinib mesylate. Vet Immunol Immunopathol.

[21] Katayama R, Huelsmeyer MK, Marr AK, Kurzman ID, Thamm DH, Vail DM (2004). Imatinib mesylate inhibits platelet-derived growth factor activity and increases chemosensitivity in feline vaccine-associated sarcoma. Cancer Chemother Pharmacol.

[22] Kiupel M, Webster JD, Kaneene JB, Miller R, Yuzbasiyan-Gurkan V (2004). The use of KIT and tryptase expression patterns as prognostic tools for canine cutaneous mast cell tumors. Vet Pathol.

[23] Dickerson EB, Marley K, Edris W, Tyner JW, Schalk V, Macdonald V (2013). Imatinib and dasatinib inhibit hemangiosarcoma and implicate PDGFR-beta and Src in tumor growth. Trans Oncol.

[24] Dubreuil P, Letard S, Ciufolini M, Gros L, Humbert M, Casteran N (2009). Masitinib (AB1010), a potent and selective tyrosine kinase inhibitor targeting KIT. PLoS One.

[25] Thamm DH, Rose B, Kow K, Humbert M, Mansfield CD, Moussy A (2012). Masitinib as a chemosensitizer of canine tumor cell lines: a proof of concept study. Vet J.

[26] Hahn KA, Oglivie G, Rusk T, Devauchelle P, Leblanc A, Legendre A (2008). Masitinib is safe and effective for the treatment of canine mast cell tumors. J Vet Int Med.

[27] Roskoski R (2015). A historical overview of protein kinases and their targeted small molecule inhibitors. Pharmacol Res.

[28] Humbert M, Casteran N, Letard S, Hanssens K, Iovanna J, Finetti P (2010). Masitinib combined with standard gemcitabine chemotherapy: in vitro and in vivo studies in human pancreatic tumour cell lines and ectopic mouse model. PLoS One.

[29] Mitry E, Hammel P, Deplanque G, Mornex F, Levy P, Seitz JF (2010). Safety and activity of masitinib in combination with gemcitabine in patients with advanced pancreatic cancer. Cancer Chemother Pharmacol.

[30] Filippi I, Naldini A, Carraro F (2011). Role of the hypoxic microenvironment in the antitumor activity of tyrosine kinase inhibitors. Curr Med Chem.

[31] Paulsson J, Ehnman M, Ostman A (2014). PDGF receptors in tumor biology: prognostic and predictive potential. Future Oncol.

[32] Uehara H, Kim SJ, Karashima T, Shepherd DL, Fan D, Tsan R (2003). Effects of blocking platelet-derived growth factor-receptor signaling in a mouse model of experimental prostate cancer bone metastases. J Natl Cancer Inst.

[33] Najy AJ, Jung YS, Won JJ, Conley-LaComb MK, Saliganan A, Kim CJ (2012). Cediranib inhibits both the intraosseous growth of PDGF D-positive prostate cancer cells and the associated bone reaction. Prostate.

[34] Shinagawa K, Kitadai Y, Tanaka M, Sumida T, Onoyama M, Ohnishi M (2013). Stroma-directed imatinib therapy impairs the tumor-promoting effect of bone marrow-derived mesenchymal stem cells in an orthotopic transplantation model of colon cancer. Int J Cancer.

[35] Urie BK, Russell DS, Kisseberth WC, London CA (2012). Evaluation of expression and function of vascular endothelial growth factor receptor 2, platelet derived growth factor receptors-alpha and -beta, KIT, and RET in canine apocrine gland anal sac adenocarcinoma and thyroid carcinoma. BMC Vet Res.

[36] Webster JD, Yuzbasiyan-Gurkan V, Miller RA, Kaneene JB, Kiupel M (2007). Cellular proliferation in canine cutaneous mast cell tumors: associations with c-KIT and its role in prognostication. Vet Pathol.

[37] Yonemaru K, Sakai H, Murakami M, Yanai T, Masegi T (2006). Expression of vascular endothelial growth factor, basic fibroblast growth factor, and their receptors (flt-1, flk-1, and flg-1) in canine vascular tumors. Vet Pathol.

[38] Asa SA, Murai A, Murakami M, Hoshino Y, Mori T, Maruo K (2012). Expression of platelet-derived growth factor and its receptors in spontaneous canine hemangiosarcoma and cutaneous hemangioma. Histol Histopathol.

[39] Sabattini S, Bettini G (2009). An immunohistochemical analysis of canine haemangioma and haemangiosarcoma. J Comp Pathol.

[40] Jensen EC (2013). Quantitative analysis of histological staining and fluorescence using ImageJ. Anat Rec.

[41] Wergin MC (2007). Effect of ionizing radiation molecular parameters in spontaneous canine tumors and canine tumor cell lines.

[42] Snyder SA, Linder K, Hedan B, Hauck ML (2011). Establishment and characterization of a canine soft tissue sarcoma cell line. Vet Pathol.

[43] Marley K, Helfand SC, Edris WA, Mata JE, Gitelman AI, Medlock J (2013). The effects of taurolidine alone and in combination with doxorubicin or carboplatin in canine osteosarcoma in vitro. BMC Vet Res.

[44] Aapro MS, Alberts DS, Salmon SE (1983). Interactions of human leukocyte interferon with vinca alkaloids and other chemotherapeutic agents against human tumors in clonogenic assay. Cancer Chemother Pharmacol.

[45] Wolfesberger B, Hoelzl C, Walter I, Reider GA, Fertl G, Thalhammer JG (2006). In vitro effects of meloxicam with or without doxorubicin on canine osteosarcoma cells. J Vet Pharmacol Ther.

[46] Viall AK, Goodall CP, Stand B, Marley K, Chappell PE, Bracha S (2014). Antagonism of serotonin receptor 1B decreases viability and promotes apoptosis in the COS canine osteosarcoma cell line. Vet Comp Oncol.

